# Human genetics and genomics as a unifying factor for harmony and progress in Africa: a report from the 12^th^ African Society of Human Genetics meeting in Bamako, Mali

**DOI:** 10.11604/pamj.2024.49.19.41189

**Published:** 2024-09-19

**Authors:** Abdoulaye Yalcouyé, Djénéba Dabitao, Oumar Samassékou, Victoria Nembaware, Fousséyni Kané, Mohamed Zahir Alimohamed, Ghada El-Kamah, Leon Mutesa, Rokhaya Ndiaye, Michele Ramsay, Seydou Doumbia, Scott Williams, Mahamadou Traoré, Ambroise Wonkam, Guida Landouré

**Affiliations:** 1 Faculté de Médecine et d’Odontostomatologie, Université des Sciences, des Techniques et des Technologies de Bamako, Bamako, Mali,; 2 Division of Human Genetics, Faculty of Health Sciences, University of Cape Town, Cape Town, South Africa,; 3 Faculté de Pharmacie, Université des Sciences, des Techniques et des Technologies de Bamako, Bamako, Mali,; 4 Department of Haematology and Blood Transfusion, Muhimbili University of Health and Allied Sciences, Dar-es-Salaam, Tanzania,; 5 Human Genetics and Genome Research Division, Centre of Scientific Excellence of Human Genetics, National Research Centre, Cairo, Egypt,; 6 Centre for Human Genetics, College of Medicine and Health Sciences, University of Rwanda, Kigali, Rwanda,; 7 Faculty of Medicine, Pharmacy and Dentistry, Cheikh Anta Diop University, Dakar, Senegal,; 8 Division of Human Genetics, School of Pathology, Faculty of Health Sciences, University of the Witwatersrand, Johannesburg, South Africa,; 9 Departments of Population and Quantitative Health Sciences, and Genetics and Genome Sciences, Cleveland Institute of Computational Biology, Case Western Reserve University, Cleveland, Ohi,; 10 McKusick-Nathans Institute of Genetic Medicine and Department of Genetic Medicine, Johns Hopkins University School of Medicine, Baltimore, Maryland, USA,; 11 Service de Neurologie, Centre Hospitalier Universitaire du Point G, Bamako, Mali

**Keywords:** Human genetics, genomics, Conference, Mali, Africa

## Abstract

Since its inception in 2003, the African Society of Human Genetics (AfSHG) has been central to the promotion of genetics research on the continent, and facilitated the networking of African researchers within Africa and abroad, thereby significantly contributing to the career development of African geneticists. The continuation of these accomplishments was stimulated by the 12^th^ international conference of AfSHG held jointly with the 1^st^ Congress of the Malian Society of Human Genetics (MSHG) in Bamako, Mali from September 18^th^ to 21^st^ 2019. The main theme of the conference was “Human Genetics and Genomics as a Unifying Factor for Harmony and Progress in Africa”. The goals of the meeting were to promote the work conducted mainly by African researchers and to contribute to scientific knowledge through genetic research. Despite challenges due to security issues in Mali, this conference attracted many scientists, including key experts in genetics and associated fields, making the conference successful scientifically and geographically. Overall, 172 delegates from 24 countries attended. Sessions on various topics relevant to Africa were held. These included the genetics of infectious diseases, cancer, and rare diseases as well as bioinformatics, pharmacogenomics, population genetics, and ethical, legal, and social issues, particularly with respect to genetic research in African populations. The need for genetic data sharing to improve research and health and the focus of actionable research for African populations was stressed throughout the meeting.

## Report

Human genetics and genomics have become central to many aspects of medical research as the understanding of how genetic variation influences disease susceptibility is increasingly important in defining risk and etiological models [1]. Over the past few decades, substantial technological advances and more efficient analytical approaches have permitted finer-scale interrogation of genetic variation. Despite these advances, Africa and African researchers have contributed relatively little to genetics/genomics research likely due to several factors including the burden of infectious diseases and the limited access to genetic testing facilities. Therefore, African researchers including those from the diaspora, and researchers interested in Africa convened in Accra, Ghana to create the African Society of Human Genetics (AfSHG) in 2003. The objectives were to provide a forum for African researchers in the field of genetics and genomics to meet, interact and collaborate, and to equip African scientists in infrastructure and practical knowledge to contribute to international research [2]. The goal was to bring to the forefront the importance of studying African populations and help develop solutions for the burden of common and rare diseases across the African continent.

**Figure 1:**
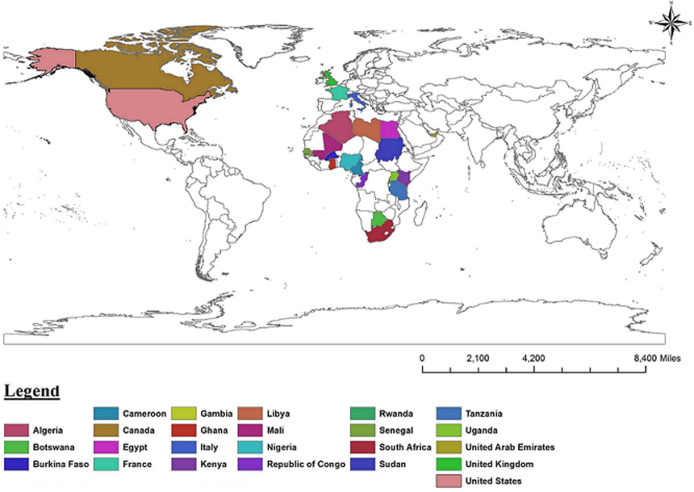
map of countries represented at the 12^th^ conference of the African Society of Human Genetics (AfSHG)

Since then the AfSHG has fostered the formation of national human genetics societies in several countries and launched initiatives to boost genetic research on the African continent, including the Human Heredity and Health in Africa (H3Africa) and Harnessing Data Science for Health Discovery and Innovation in Africa (DS-I Africa) [2-5]. The 12^th^ conference of the AfSHG was co-organized with the first congress of the Malian Society of Human Genetics (MSHG) and was held September 18-21, 2019 in Bamako. The main theme of the conference was “Human Genetics and Genomics as a Unifying Factor of Harmony and Progress in Africa”. One hundred seventy-two delegates from 24 countries attended this high-level scientific meeting (Figure 1). This meeting provided an excellent opportunity for Malian scientists and all the attendees to learn about the latest updates and knowledge in genetics. Special sessions were dedicated to several research topics including ethical, legal, and social issues.

### Highlights of the 12^th^ conference

**Young Investigators Forum (YIF):** as per the AfSHG tradition, a day prior to the main conference was used to give young researchers the opportunity to present their work in humans genetics [6]. The Young Investigator Forum was held in the International Center for Conferences of Bamako (CICB) on September 18, 2019, with nearly 200 participants. Overall, 26 oral presentations and 15 posters were scheduled for that day. At this forum, different aspects covering the genetics of rare and common diseases including infectious diseases, cancers, hematological diseases, and hereditary neurological diseases were discussed by young researchers. In addition, a special workshop on scientific writing was facilitated by Taahira Goolam Hoosen, an expert from the University of Cape Town in South Africa. This forum was organized by the young researchers themselves under the supervision of their mentors. All the sessions were chaired by young researchers who are emerging as junior experts in different aspects of genetics. This day was a testament to the AfSHG intention to facilitate intellectual exchange and network between young researchers from Africa and prepare them to be leaders of the AfSHG and genetic research on the continent. A talk was given by Prof. Ambroise Wonkam, the current president of the AfSHG entitled “How to inspire and improve the next generation of African scientists”? Access to this forum was free to allow participation by the maximum of young professionals, so they could benefit from this unique opportunity to network with other young researchers and be in touch with current science.

**Main conference:** the main conference took place from September 19 to 21^st^ in Bamako. During these three days, researchers from Africa, Europe, and America shared their scientific findings covering most, if not all, aspects of human genetics. The opening ceremony was chaired by the Ministry of Higher Education and Research. In total, three keynote speakers, 15 invited speakers and 31 speakers selected via abstract review presented their work in seven sessions as follows: after the inaugural ceremony, Prof. Muntaser Ibrahim (Department of Molecular Biology, University of Khartoum, Sudan) gave the first keynote address on the topic of “Human genome as foreteller”.

**Population and non-communicable diseases:** during this session, Prof Emile Chimusa (University of Cape Town, South Africa) described the opportunities and challenges of conducting genetic studies in admixed populations. How the diversity of the African population aided the discovery of some variants driving susceptibility for developing carotid intima and media thickness was presented by Mr Palwende Romuald Boua *(Institut de Recherche en Sciences de la Santé, Burkina Faso)*. Other population-based studies indicated the difficulties existing in genetic and genomic research in the African context.

**Genetics of infectious diseases:** before this session, a plenary session was given by Prof Abdoulaye Djimdé (Faculty of Pharmacy, USTTB, Mali) on the genetic diversity of both parasites responsible for malaria and the human host, particularly in an African population. This talk highlighted how little was known regarding the genetic diversity of the malaria parasites. Prof Youssef Idaghdour, Center for Genomics and Systems Biology New York University Abu Dhabi, United Arab Emirates, identified both genetic and environmental determinants of host response to malaria that remains a major public health concern for Africans. Dr Mogomotsi S. Matshaba (Botswana-Baylor Children's Clinical Center of Excellence) spoke on the relevant historical perspective on the evolution of TB and HIV in Africa, which remain a public health concerns in many African countries.

**Genomic medicine:** before this session, Prof Michele Ramsay (University of the Witwatersrand, South Africa) gave a plenary talk on the use of Pan-African GWAS to detect associations with previously associated lead single nucleotide variants (SNVs) and novel SNVs at known gene loci for lipid traits. The highly associated novel locus, GATB was also discussed in relation to lipoprotein-cholesterol (LDL-C) in African populations. Prof Ambroise Wonkam (University of Cape Town, South Africa) spoke on “Genetic Medicine of African populations”, during which he highlighted the necessity of conducting genetic studies in Africa because of its unique genetic diversity and its potential contribution to global efforts to understand human traits.

**Cancer and developmental genetics:** the speakers developed themes including the role of genetics in growth disorders and the particular challenges in Africa (Prof Léon Mutesa, University of Rwanda, Rwanda). Dr Oumar Samassékou´s (USTTB, Mali) talk on “The 3D Nuclear Organization of Telomere as a Genomic Exploration Tool in Cancer” revealed that the 3D telomere profile can help differentiate pathological groups of cancers, predict progression-free survival and overall survival.

**Genetics of rare diseases and hemoglobinopathies:** in this session, Prof Ghada El-Kamah (University of Cairo, Egypt) advocated for the establishment of a genodermatoses registry and a clinical database that is currently absent in most developing countries. Dr Ahmed Yasir from UK discussed the cellular pathogenesis of rare variants that increase risk for neurodevelopmental disorders. Other relevant diseases including sickle cell anemia and novel clinical and/or genetic variants implicated in several hereditary neurological conditions were also discussed, confirming the role of genetic heterogeneity in an African population.

**Ethical, social, and legal aspects:** this session recognized ethics as a key component of research, especially genetic research. It is not acceptable to conduct proper biomedical research without social, legal ethical considerations. In this session, experts in research ethics developed ethical principles that should guide clinical and genetic research with human subjects enrolled in resource-limited settings by considering socio-cultural context. Prof Coulibaly Souleymane (USTTB, Mali) Mali spoke on “Problems of returning the genetic testing results at the Teaching Hospital of Point G” with emphasis on the particular social and cultural contexts in Mali that make the work more difficult.

**Pharmacogenomics and bioinformatics:** three invited speakers and two selected abstracts including one from a young investigator were included in this session. That current research in genetics depends heavily on bioinformatics tools was highlighted by Profs Collet Dandara and Seydou Doumbia.

**Perspectives:** the AfSHG provides an important venue for African researchers in the field of genetics to meet and exchange their ideas and experiences. This conference was successfully organized in Mali despite the security issues and the travel restrictions from many European and American countries. This meeting allowed exchanges between scientists even from the same country, established foundations for collaboration between researchers, and provided a framework for harmony by bringing together researchers on the continent with those from abroad. All these aspects highlight the progress of genetic research on the African continent. In the absence of extensive research on the genetic contribution to diseases in many African populations, it would not be possible to develop appropriate genetic services or to lay groundwork for the implementation of precision medicine approaches on the continent. As many African countries do not have human genetics as a stand-alone discipline as reported by Wonkam *et al*. [7] we must be creative in including this discipline in activities and existing structures in our tertiary establishments, hospitals and health care. Such effort will promote genetics for future patient care. Despite the limited resources and the difficulties experienced on the African continent, the AfSHG continues to successfully promote genetic research in Africa by disseminating human genetics research in Africa, establishing a mentorship network providing educational resources, including the development of appropriate technology transfer, and providing advocacy for human genetic research in Africa, and encouraging collaborative research [2].

## Conclusion

With the recent advances of genetic investigative tools, genetic studies are becoming more accessible. During this conference, it was highlighted that genetic studies are necessary to understand pathogenesis of most, if not all diseases, and that studies conducted in African populations where genetic diversity is more extensive than in other human populations is critical. The 12^th^ international conference of the AfSHG was a high-level educational and scientific meeting. With experts in genetics who came from around the world, it served as an excellent platform for researchers to network and share their findings. Many junior and senior African scientists had the opportunity to present their data to an expert audience. Training of the next generation of African scientists in the field of data science and bioinformatics was also discussed.
